# Medication Non-adherence and Condomless Anal Intercourse Increased Substantially During the COVID-19 Pandemic Among MSM PrEP Users: A Retrospective Cohort Study in Four Chinese Metropolises

**DOI:** 10.3389/fmed.2022.738541

**Published:** 2022-04-29

**Authors:** Yangyang Gao, Qinghai Hu, Sequoia I. Leuba, Le Jia, Hongyi Wang, Xiaojie Huang, Yaokai Chen, Hui Wang, Jing Zhang, Zhenxing Chu, Lukun Zhang, Zixin Wang, Hong Shang, Junjie Xu, CROPrEP Study Team, Xiaoqing He

**Affiliations:** ^1^National Health Commission Key Laboratory of Acquired Immunodeficiency Syndrome (AIDS) Immunology (China Medical University), National Clinical Research Center for Laboratory Medicine, The First Affiliated Hospital of China Medical University, Shenyang, China; ^2^Key Laboratory of AIDS Immunology, Chinese Academy of Medical Sciences, Shenyang, China; ^3^Key Laboratory of AIDS Immunology of Liaoning Province, Shenyang, China; ^4^Collaborative Innovation Center for Diagnosis and Treatment of Infectious Diseases, Hangzhou, China; ^5^Department of Epidemiology, University of North Carolina at Chapel Hill, Chapel Hill, NC, United States; ^6^Center for Infectious Diseases, Beijing Youan Hospital, Capital Medical University, Beijing, China; ^7^Chongqing Public Health Medical Center, Chongqing, China; ^8^Department of Infectious Diseases, National Clinical Center for Infectious Diseases, Third People's Hospital of Shenzhen, Second Affiliated Hospital of Southern University of Science and Technology, Shenzhen, China; ^9^Faculty of Medicine, The Jockey Club School of Public Health and Primary Care, The Chinese University of Hong Kong, Hong Kong, Hong Kong SAR, China

**Keywords:** COVID-19, pre-exposure prophylaxis (PrEP), adherence–compliance–persistence, men who have sex with men (MSM), condomless anal intercourse (CAI)

## Abstract

**Background:**

The coronavirus disease (COVID-19) pandemic has impacted HIV prevention strategies globally. However, changes in pre-exposure prophylaxis (PrEP) adherence and HIV-related behaviors, and their associations with medication adherence among men who have sex with men (MSM) PrEP users remain unclear since the onset of the COVID-19 pandemic.

**Methods:**

A Retrospective Cohort Study of HIV-negative MSM PrEP users was conducted in four Chinese metropolises from December 2018 to March 2020, assessing the changes in PrEP adherence and HIV-related behaviors before and during the COVID-19. The primary outcome was poor PrEP adherence determined from self-reported missing at least one PrEP dose in the previous month. We used multivariable logistic regression to determine factors correlated with poor adherence during COVID-19.

**Results:**

We enrolled 791 eligible participants (418 [52.8%] in daily PrEP and 373 [47.2%] in event-driven PrEP). Compared with the data conducted before the COVID-19, the proportion of PrEP users decreased from 97.9 to 64.3%, and the proportion of poor PrEP adherence increased from 23.6 to 50.1% during the COVID-19 [odds ratio (*OR*) 3.24, 95% confidence interval (*CI*) 2.62–4.02]. While the percentage of condomless anal intercourse (CAI) with regular partners (11.8 vs. 25.7%) and with casual partners (4.4 vs. 9.0%) both significantly increased. The proportion of those who were tested for HIV decreased from 50.1 to 25.9%. Factors correlated with poor PrEP adherence during the COVID-19 included not being tested for HIV (adjusted odds ratio [a*OR*] = 1.38 [95% *CI*: 1.00, 1.91]), using condoms consistently with regular partners (vs. never, a*OR* = 2.19 [95% *CI*: 1.16, 4.13]), and being married or cohabitating with a woman (vs. not married, a*OR* = 3.08 [95% *CI*: 1.60, 5.95]).

**Conclusions:**

Increased poor PrEP adherence and CAI along with the decrease in HIV testing can lead to an increase in HIV acquisition and drug resistance to PrEP. Targeted interventions are needed to improve PrEP adherence and HIV prevention strategies.

## Introduction

Since the declaration of the coronavirus disease 2019 (COVID-19) pandemic by the World Health Organization (WHO) on 11 March 2020, social distancing has interrupted hospital-based HIV prevention methods, HIV care, and testing services, particularly among men who have sex with men (MSM) (1, 2). Almost one quarter (23%) of new HIV infections globally in 2019 were among MSM, and the percentage of incident HIV infections among MSM was even higher in the Asia-Pacific region (3). The HIV incidence among Chinese MSM has increased from 3.24/100 PY (95% *CI*: 2.81–3.74) in 2005–2008 to 5.95/100 PY (95% *CI*: 5.37–6.56) in 2012–2018 (4). New strategies to prevent HIV transmission, especially those that are effective despite social distancing, are needed to address the increasing HIV epidemic among Chinese MSM.

Pre-exposure prophylaxis (PrEP) is an innovative and effective biomedical HIV prevention strategy for people at high risk of HIV infection (5). PrEP is a medication that is usually taken daily or event-driven to prevent HIV transmission and is frequently tenofovir/emtricitabine (TDF/FTC). If used with optimal adherence, PrEP is highly effective at preventing HIV transmission (6, 7). However, if adherence is <40%, PrEP is no longer protective against HIV transmission (8). Since the onset of the COVID-19 pandemic, few studies have assessed PrEP adherence but several have reported that the number of PrEP users has decreased significantly (9, 10). In the United Kingdom, Belgium, and Australia, there have been 80.0, 47.0, and 41.8% reductions in HIV PrEP users after the outbreak of COVID-19, respectively (11–13). In addition to decreasing the use of PrEP, changes in HIV-related sexual behaviors and increased barriers to accessing HIV prevention and testing services during the COVID-19 pandemic could lead to an increase in HIV acquisition (9, 11, 14). Little is known about possible changes in PrEP adherence and HIV-related sexual and testing behaviors from before to during the COVID-19 pandemic.

This study was based on the China Real-world Oral Intake of PrEP (*CROPrEP*) project, which is an ongoing multi-center, real-world trial of HIV PrEP among Chinese MSM to assess the effectiveness and adherence of daily or event-driven PrEP (15). Participants would complete five follow-up visits that included an online questionnaire and a clinic visit at 4, 12, 24, 36, and 48 weeks after enrollment. After clinical evaluations and HIV laboratory testing, participants received TDF/FTC tablets to use as PrEP. We, thus, investigated PrEP adherence and HIV-related sexual and testing behaviors among Chinese MSM PrEP users and determining factors correlated with poor adherence before and during the COVID-19 outbreak. Our findings will help researchers develop interventions to maintain and support PrEP use during the pandemic.

## Methods

### Study Design

The study recruited 791 MSM aged 16–65 years from four major Chinese cities (Shenyang, Beijing, Shenzhen, and Chongqing) from December 2018 to March 2020. In this study, we invited participants to complete two online self-administered surveys. The aim of this study was to determine changes in PrEP adherence and HIV-related and testing behaviors among PrEP-using Chinese MSM from before to during the COVID-19 outbreak. The median interval between the 4-week visit and this additional online survey was 28 weeks, and the interquartile range (IQR) was 24–36 weeks.

### Participants

The inclusion criteria for this additional online survey were the following: (1) participants of the *CROPrEP* project, (2) screened to be HIV-negative at the most recent follow-up visit, and (3) had not yet completed the final follow-up visit at Week 48. We excluded those who did not complete a Week 4 follow-up visit as data from this visit were used to provide information from before the COVID-19 outbreak.

### Data Collection

This additional online survey assessing changes in PrEP adherence and HIV-related sexual and testing behaviors due to the COVID-19 pandemic was developed by a panel consisting of HIV epidemiologists, clinicians, and MSM-serving community-based organization members. This questionnaire was then pilot-tested among 15 MSM volunteers who were not *CROPrEP* participants and was revised based on their comments. A link to this additional online questionnaire was shared among all participants in *CROPrEP* by using the WeChat (i.e., a popular social media platform) group. If participants did not complete the survey within 24 h, project staff reminded them of the invitation *via* a phone call or a text message. Participation in this additional online survey was voluntary, refusal to participate had no effect on their participation in the *CROPrEP* project, and data were confidential and used only for research purposes. Each individual account was allowed to access the online questionnaire only once to avoid duplicate responses (refer to Supplementary Appendix 1).

### Measures

Before the COVID-19, measures were determined from the 4-week follow-up visit for *CROPrEP*, conducted between October 2018 and November 2019, and during the COVID-19, measures were determined from the additional online survey conducted between February and March 2020. Baseline background characteristics assessed included type of PrEP regimen, age, education, monthly income, marital status, sexual identity, and lockdown restrictions experience (during the COVID-19 survey only). We asked about HIV-related sexual behaviors, such as primary locations to seek male sexual partners, the number and types of male sexual partners, frequency of sexual acts, frequency of condomless anal intercourse (CAI) with specific types of male sexual partners, and sexualized drug use. For HIV testing behavior, we asked about the self-perceived risk of HIV infection, HIV test behavior in the past month, location of the HIV test [i.e., facility-based HIV testing or HIV self-testing (HIVST)], and self-assessment of the frequency of HIV testing compared with pre-COVID-19 (during the COVID-19 survey only). To assess changes, we also asked whether they had experienced lockdown restrictions due to COVID-19, how frequently were they concerned about the COVID-19 pandemic (i.e., never, sometimes, often, and always), or whether they had delayed a scheduled follow-up visit for the *CROPrEP* project (refer to Supplementary Appendix 2).

Male sexual partners were defined as regular (i.e., those who were in a stable relationship and did not involve transactional sex) or casual (i.e., those who were not in a stable relationship and did not involve transactional sex). Sexualized drug use was defined as using any of the following drugs during sexual relations in the previous month: rush poppers (alkyl nitrites), cocaine, methamphetamine, ketamine, and bath salts. Having delayed a scheduled follow-up visit for the *CROPrEP* project was defined as attending a follow-up visit after the previously scheduled appointment date by 7 days or more.

### Outcome

Self-reported PrEP adherence was defined based on a comprehensive evaluation of the self-reported missed PrEP doses and sexual behaviors. Poor adherence was defined as the following: (1) missing doses among daily PrEP users in the past month and (2) missing doses among event-driven PrEP users if they had sexual behaviors in the past month. If not defined as having poor adherence, participants were defined as having good adherence during the COVID-19 (16).

### Statistical Analysis

We analyzed the demographics and HIV-related behaviors of Chinese MSM PrEP users using frequencies and percentages. We then used the generalized estimating equation (GEE model-Binary logistic regression) to analyze the changes in adherence and HIV-related behaviors sexual and testing behaviors, and PrEP use in the past month from before (using data from the 4-week follow-up visit from *CROPrEP*) and during the COVID-19 outbreak (using data collected from the additional online survey during COVID-19). We then assessed factors correlated with poor adherence using univariable and multivariable logistic regression models adjusted for age, education, and monthly income. A two-tailed value of *p* below 0.05 and between 0.05 and 0.10 was considered statistically significant and marginally significant, respectively. All statistical analyses were performed using SPSS™ software version 25.0 (IBM Corp, Armonk, NY, US).

### Ethical Review

This study was reviewed and approved by the Medical Science Research Ethics Committee of the First Affiliated Hospital of China Medical University ([2018]2015-139-5) and was registered with the Chinese Clinical Trial Registry (ChiCTR-IIN-17013762). After providing online informed consent, eligible participants were asked to complete the additional questionnaire and had the opportunity to review and modify their responses. All data and informed consent were password-protected and stored in a secure server, and only the principal investigator had access to the database. Each participant was compensated $4.20 (30 Yuan) after completing the additional online survey (refer to Supplementary Appendix 3).

## Results

### Baseline Characteristics

**Figure 1:**
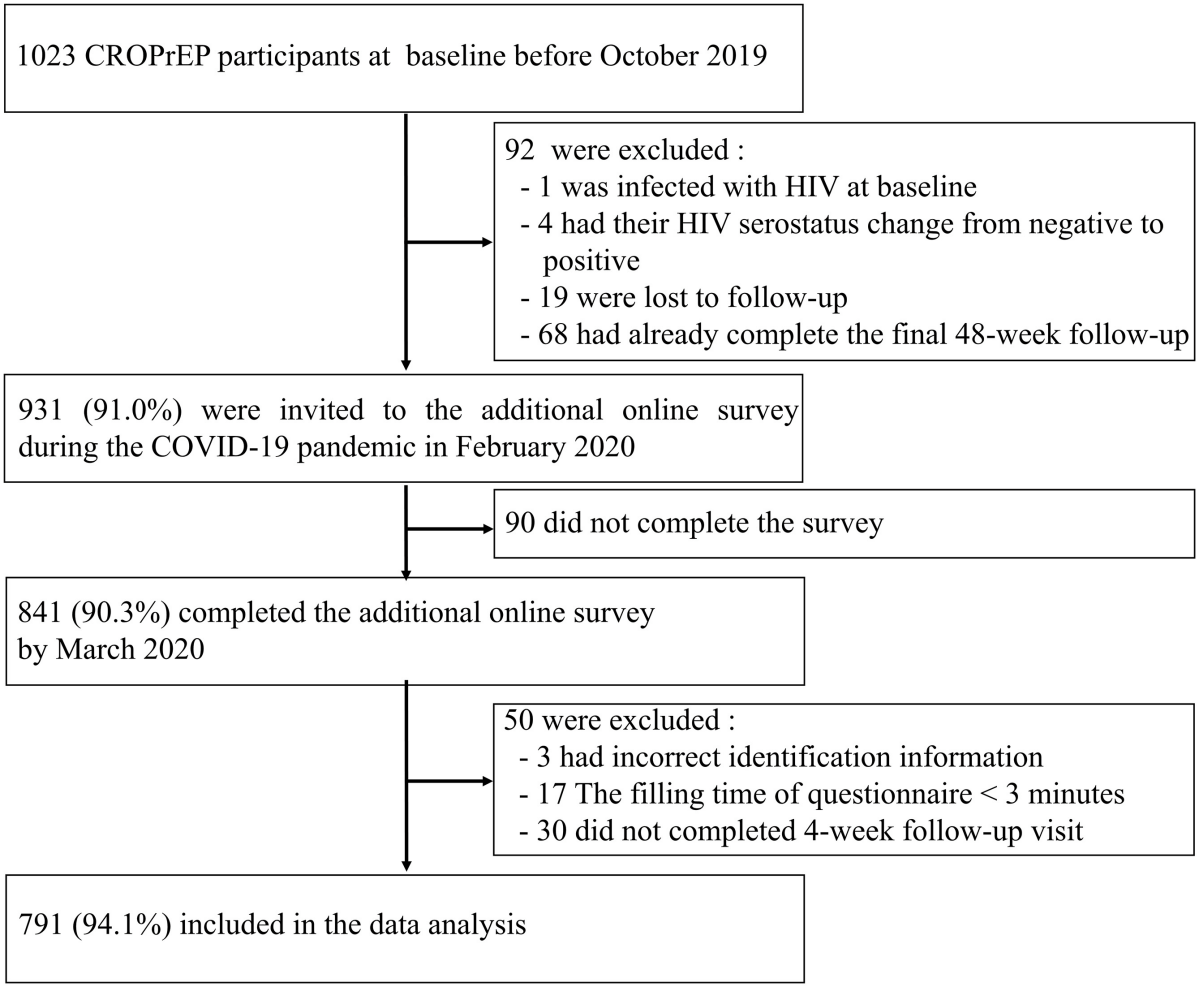
Study profile.

Before the COVID-19 outbreak, 1,023 MSM were enrolled in the *CROPrEP* trial, and 931 (91.0%) were invited to participate in this survey. During the COVID-19 pandemic, 841 (90.3%) invited participants completed the online survey, and of these participants, 791 (94.1%) were included in the data analysis (300 in Shenyang, 362 in Beijing, 61 in Shenzhen, and 68 in Chongqing) (refer to Figure 1). The median age of participants was 30 years (IQR: 26–36 years), 79.6% (630/791) had an education level of college and above, 38.1% (301/791) had a monthly income of 2,001–6,000 Yuan (~$310–929), and 81.0% (641/791) self-described their sexual identity as a homosexual. In response to COVID-19, 93.6% (740/791) of participants reported experiencing COVID-19 lockdown restrictions, such as physical distancing, community restrictions, and the banning of indoor gatherings (refer to Table 1).

**Table 1:** Characteristics of Chinese MSM PrEP users during the COVID-19 pandemic (*N* = 791).

**Characteristic**	**Participants (*n*, %)**
**PrEP regimen**	
Daily	418 (52.8)
Event-driven	373 (47.2)
**Age (years)**	
18-24	130 (16.4)
25-39	534 (67.5)
40-65	127 (16.1)
**Education**	
High school and below	161 (20.4)
College and above	630 (79.6)
**Average monthly income (RMB, Yuan)**	
<2,000	129 (16.3)
2,001-6,000	301 (38.1)
6,001-10,000	153 (19.3)
More than 10,000	208 (26.3)
**Marital status**	
Not married	499 (63.1)
Married or cohabitating with a woman	61 (7.7)
Cohabitating with a male	203 (25.7)
Divorced, separated, or widowed	28 (3.5)
**Sexual identity**	
Homosexual	641 (81.0)
Bisexual	126 (15.9)
Heterosexual	3 (0.4)
Not sure	21 (2.7)
Experienced lockdown restrictions in response to COVID-19^*^	
Yes	740 (93.6)
No	51 (6.4)

*Data are from the additional online survey administered during the COVID-19 pandemic*.

*MSM, men who have sex with men; PrEP, pre-exposure prophylaxis; COVID-19, Coronavirus disease 2019*.

* *Lockdown restrictions include physical distancing of two meters when individuals have to leave their homes, community restrictions, and the banning of indoor gatherings*.

### Changes in HIV-Related Sexual Behavior, HIV Testing, and PrEP Adherence From Before to During the COVID-19 Pandemic

Sexual acts, sexualized drug use, HIV testing, and PrEP use and adherence sharply decreased as more participants had no partner during the pandemic. The percentage of participants who had regular or no male sexual partners in the past month greatly increased from 33.4% (264/791) before to 74.0% (585/791) during COVID-19, and the percentage of those who used the internet as their primary location to seek male sexual partners dropped from 60.4% (478/791) before to 25.3% (200/791) during COVID-19. In addition, sexual activity, such as frequency of sexual acts, having 2 or more male sexual partners and having either a regular or casual male sexual partner greatly decreased from before to during COVID-19. However, the proportion of CAI with regular (11.8–25.7%) and casual (4.4–9.0%) male sexual partners increased during COVID-19. Sexualized drug use sharply decreased from 39.8% (315/791) before to 24.1% (191/791) during COVID-19. HIV testing behaviors sharply decreased from 50.1% (396/791) of participants having been tested for HIV in the past month before COVID-19 to 25.9% (205/791) during COVID-19, including HIVST (34.5% (273/791) before to 20.7% (164/791) during COVID-19) or through a facility [26.9% (213/791) before to 6.4% (51/791) during COVID-19]. Prevention of HIV also decreased as PrEP use dropped from 97.9% (774/791) before to 64.5% (510/791) and poor PrEP adherence increased from 23.6% (187/791) before to 50.1% (396/791) during COVID-19. Participants also delayed a scheduled follow-up visit for the *CROPrEP* trial more during COVID-19 (14.5%, 115/791) than before (10.0%, 79/791) (refer to Table 2).

**Table 2:** Changes in sexual behaviors, HIV testing, and PrEP adherence among during compared to before the COVID-19 (*N* = 791).

	**Before COVID−19^**a**^**	**During COVID−19**	**Odds ratio**	***P*-value**
	***n* (%)**	***n* (%)**	**(95% CI)**	
**HIV–related sexual behaviors in the past month**				
**Primary location to seek male sexual partners**				
Internet	478 (60.4)	200 (25.3)	0.22 (0.18, 0.27)	<0.001
Park/Bathroom/Club	49 (6.2)	6 (0.8)	0.12 (0.05, 0.27)	<0.001
Had regular or no male sexual partners	264 (33.4)	585 (74.0)	5.68 (4.60, 701)	<0.001
**Frequency of sexual acts** ^**b**^				
More than once a week	413 (52.2)	125 (15.8)	0.17 (0.14, 0.21)	<0.001
Once a week or less than once a week	321 (40.6)	224 (28.3)	0.58 (0.47, 0.72)	<0.001
No sex	57 (7.2)	442 (55.9)	16.31 (12.04, 22.10)	<0.001
**Sexual partners in the past month**				
Had two or more male sexual partners	536 (67.8)	136 (17.2)	0.10 (0.08, 0.12)	<0.001
Had regular male sexual partners	467 (59.0)	249 (31.5)	0.32 (0.26, 0.39)	<0.001
Had casual male sexual partners	317 (40.1)	134 (16.9)	0.30 (0.24, 0.38)	<0.001
**Condom use with regular male sexual partners** ^**c**^				
Consistently	220 (47.1)	121 (48.6)	1.05 (0.79, 1.44)	0.745
Most or sometimes	192 (41.1)	64 (25.7)	0.05 (0.36, 0.70)	<0.001
Never	55 (11.8)	64 (25.7)	2.61 (1.76, 2.87)	<0.001
**Condom use with casual male sexual partners** ^**c**^				
Consistently	167 (52.7)	74 (55.2)	1.13 (0.75, 1.71)	0.566
Most or sometimes	136 (42.9)	48 (35.8)	0.73 (0.348, 1.11)	0.143
Never	14 (4.4)	12 (9.0)	2.09 (0.92, 4.72)	0.077
**Sexualized drug use** ^**d**^	315 (39.8)	191 (24.1)	0.21(0.17, 0.26)	<0.001
**HIV testing behaviors in the past month**				
Had HIV test	396 (50.1)	205 (25.9)	0.35 (0.28, 0.43)	<0.001
Had HIV test through HIVST	273 (34.5)	164 (20.7)	0.50 (0.40, 0.61)	<0.001
Had facility–based HIV testing	213 (26.9)	51 (6.4)	0.19 (0.13, 0.26)	<0.001
**PrEP status in the past month**				
**PrEP regimen**				
Daily	410 (51.8)	418 (52.8)	1.04 (0.86, 1.26)	0.682
Event-driven	381 (48.2)	373 (47.2)	0.96 (0.79, 1.17)	0.682
**Used PrEP**	774 (97.9)	510 (64.5)	0.04 (0.02, 0.07)	<0.001
**Poor PrEP adherence**	187 (23.6)	396 (50.1)	3.24 (2.62, 4.02)	<0.001
**Delayed scheduled** ***CROPrEP*** **follow–up visit**	79 (10.0)	115 (14.5)	1.54 (1.13, 2.08)	0.006
**Self–perceived risk of HIV infection**				
No risk	160 (20.2)	367 (46.4)	3.41 (2.72, 4.28)	<0.001
Low risk (<25%)	419 (53.0)	315 (39.8)	0.59 (0.48, 0.72)	<0.001
Moderate risk (25–49%)	142 (18.0)	68 (8.6)	0.43 (0.32, 0.58)	<0.001
High risk (50–75%)	52 (6.6)	24 (3.0)	0.45 (0.27, 0.72)	0.001
Very high risk (>75%)	18 (2.3)	17 (2.1)	0.94 (0.49, 1.82)	0.862

*ALL data are among Chinese MSM PrEP users*.

*COVID-19, coronavirus disease 2019; MSM, men who have sex with men; PrEP, pre-exposure prophylaxis; HIVST, HIV self-testing*.

*CROPrEP, China Real-world Oral Intake of PrEP*.

a *Before COVID-19 estimates were determined from the 4th week follow-up visit of the China Real-world Oral Intake of PrEP (CROPrEP) project conducted between October 2018 to November 2019*.

b *Sexual acts included receptive anal intercourse, insertive anal intercourse, and oral intercourse*.

c *Only people with regular or casual partners answered the question*.

d *Sexualized drug use in the past month included use of rush poppers (alkyl nitrites), cocaine, methamphetamine, ketamine, and bath salts before or during sexual activity*.

In the generalized estimating equations model, there was a significant decrease in the odds of using PrEP (odds ratio (*OR*) = 0.04 [95% *CI*: 0.02, 0.07], *p* < 0.001) and a significant increase in the odds of self-reporting missing at least one PrEP dose (*OR* = 3.24 [95% *CI*: 2.62, 4.02], *p* < 0.001) and in the odds of delaying a scheduled *CROPrEP* follow-up visit (*OR* = 1.54 [95% *CI*: 1.13, 2.08], *p* = 0.006) compared during to before the COVID-19 pandemic. Additionally, there were significant reductions in the odds of having been tested for HIV in the past month (*OR* = 0.35 [95% *CI*: 0.28, 0.43], *p* < 0.001). In concordance with the decrease in HIV-related sexual behavior, there was a significant increase in the odds of self-perceiving risk of HIV infection as compared with no risk (*OR* = 3.41 [95% *CI*: 2.72, 4.28], *p* < 0.001). By contrast, among those still having sexual behavior during the COVID-19, there were significant increases in the odds of having CAI with regular male sexual partners (*OR* = 2.61 [95% *CI*: 1.76, 2.87], *p* < 0.001) and marginal increases in the odds of having CAI with casual male sexual partners (*OR* = 2.09, 95% *CI*: 0.92, 4.72, *p* = 0.077) compared to before COVID-19 (refer to Table 2).

### Factors Correlated With Poor PrEP Adherence During the COVID-19 Pandemic

About half of the participants (396/791, 50.1%) were defined as having poor PrEP adherence during the COVID-19. After adjusting for age, education, and income, the following factors over the past month were associated with increased odds of poor PrEP adherence during the COVID-19 pandemic: being married or cohabitating with a woman (compared with not married: a*OR* = 3.08 [95% *CI*: 1.60, 5.95], *p* < 0.001), using condoms consistently with regular male sexual partners (compared with never using condoms: a*OR* = 2.19 [95% *CI*: 1.16, 4.13], *p* = 0.016), and often concerned about the COVID-19 pandemic (compared with always concerned: a*OR* = 1.45 [95% *CI*: 1.07, 1.97], *p* = 0.017). Knowing the HIV status of regular male sexual partners (a*OR* = 1.44 [95% *CI*: 0.98, 2.11], *p* = 0.065) and not having been tested for HIV in the previous month (compared with have been tested for HIV: a*OR* = 1.38 [95% *CI*: 1.00–1.91], *p* = 0.050) were associated with marginally higher odds of poor PrEP adherence. There was no difference in the odds of poor or good PrEP adherence based on the PrEP dosing regimen (event-driven vs. daily: a*OR* = 1.09 [95% *CI*: 0.82, 1.45], *p* = 0.560) (refer to Table 3).

**Table 3:** Factors correlated with poor adherence to PrEP among Chinese MSM PrEP users during the COVID-19 (*N* = 791).

	**Poor adherence**	**Good adherence**	**Odds ratio**	**Adjusted odds ratio**	***P*-value**
	***n* = 396, *n* (%)**	***n* = 395, *n* (%)**	**(95% CI)**	**(95% CI)^**a**^**	
**PrEP regimen**					
Event-driven	192 (48.5)	181 (45.8)	1.11 (0.84, 1.47)	1.09 (0.82, 1.45)	0.560
Daily	204 (51.5)	214 (54.2)	Reference	Reference	
**Marital status**					
Married or cohabitating with a woman	45 (11.4)	16 (4.1)	3.10 (1.70, 5.63)	3.08 (1.60, 5.95)	0.001
Cohabitating with male	98 (24.7)	105 (26.6)	1.03 (0.74, 1.43)	1.00 (0.72, 1.40)	0.973
Divorced, separated, or widowed	15 (3.8)	13 (3.3)	1.36 (0.64, 2.88)	1.43 (0.64, 3.18)	0.382
Not married	238 (60.1)	261 (66.1)	Reference	Reference	
**Primary location to seek male sexual partners**					
No male sexual partner	197 (49.7)	173 (43.8)	1.48 (1.05, 2.09)	1.47 (1.04, 2.09)	0.030
Park/Bathroom/Club	3 (0.8)	3 (0.8)	1.30 (0.26, 6.59)	1.02 (0.20, 5.26)	0.985
Had regular male sexual partners	109 (27.5)	106 (26.8)	1.34 (0.91, 1.97)	1.27 (0.86, 1.88)	0.224
Internet^b^	87 (22.0)	113 (28.6)	Reference	Reference	
**HIV–related sexual behaviors in the past month**					
**Had two or more male sexual partners**					
Yes	65 (16.4)	71 (18.0)	0.90 (0.62, 1.30)	0.83 (0.57, 1.20)	0.333
No	331 (83.6)	324 (82.0)	Reference	Reference	
**Had regular male sexual partners**					
Yes	130 (32.8)	119 (30.1)	1.13 (0.84, 1.53)	1.07 (0.79, 1.45)	0.665
No	266 (67.2)	276 (69.9)	Reference	Reference	
**Had casual male sexual partners**					
Yes	61 (15.4)	73 (18.5)	0.80 (0.55, 1.17)	0.79 (0.55, 1.15)	0.224
No	335 (84.6)	322 (81.5)	Reference	Reference	
**Condom use with regular male sexual partners**					
Consistently	71 (54.6)	50 (42.0)	2.22 (1.19, 4.11)	2.19 (1.16, 4.13)	0.016
Most or sometimes	34 (26.2)	30 (25.2)	1.77 (0.88, 3.57)	1.74 (0.84, 3.61)	0.135
Never	25 (19.2)	39 (32.8)	Reference	Reference	
**Condom use with casual male sexual partners**					
Consistently	34 (55.7)	40 (54.8)	0.85 (0.25, 2.88)	0.96 (0.28, 3.35)	0.951
Most or sometimes	21 (34.4)	27 (37.0)	0.78 (0.22, 2.76)	0.82 (0.23, 3.00)	0.769
Never	6 (9.8)	6 (8.2)	Reference	Reference	
**Know the HIV status of regular male sexual partners**					
Yes	95 (39.4)	72 (30.9)	1.46 (1.00, 2.13)	1.44 (0.98, 2.11)	0.065
No	146 (60.6)	161 (69.1)	Reference	Reference	
**HIV testing behaviors in the past month**					
**Had HIV test**					
No	304 (76.8)	282 (71.4)	1.32 (0.96, 1.82)	1.38 (1.00, 1.91)	0.050
Yes	92 (23.2)	113 (28.6)	Reference	Reference	
**Self–assessment of the frequency of HIV testing**					
**compare to pre-COVID-19**					
Increase	6 (1.5)	7 (1.8)	0.93 (0.31, 2.79)	0.87 (0.29, 2.66)	0.809
Decrease	111 (28.0)	86 (21.8)	1.40 (1.00, 1.93)	1.46 (1.05, 2.03)	0.026
No change	279 (70.5)	302 (76.5)	Reference	Reference	
**Concerned about the COVID−19 pandemic** ^c^					
Never	2 (0.5)	1 (0.3)	2.52 (0.23, 28.09)	2.90 (0.25, 33.44)	0.393
Sometimes	59 (14.9)	44 (11.1)	1.69 (1.08, 2.65)	1.74 (1.11, 2.75)	0.017
Often	193 (48.7)	171 (43.3)	1.42 (1.05, 1.92)	1.45 (1.07, 1.97)	0.017
Always	142 (35.9)	179 (45.3)	Reference	Reference	

*COVID-19, coronavirus disease 2019; MSM, men who have sex with men; PrEP, pre-exposure prophylaxis*.

a *Adjusted odds ratios were obtained through multivariable analysis and were adjusted for age, education, and monthly income*.

b *Internet include geosocial networking applications including WeChat, QQ, Blued or Jacked*.

c *Concerned about the COVID-19 pandemic was defined as how often obtaining information about COVID-19 through social media and other material proactively*.

## Discussion

In this retrospective cohort study in China, we found a dramatic increase in poor PrEP adherence along with decreases in HIV testing, the number of male sexual partners, and sexual acts during the COVID-19 pandemic. Factors correlated with the increased odds of poor PrEP adherence during COVID-19 included not having an HIV test conducted in the last month, using condoms consistently with regular male sexual partners, and being married or cohabitating with a woman. Among those who had regular or casual male sexual partners, the percentage of those who had CAI substantially increased during COVID-19 compared with the percentage before the pandemic. This may result in an increased risk of HIV infection and drug resistance in this sexually active group during the pandemic. Thus, public health officials should promote safer sex behaviors among those who are sexually active during COVID-19. In addition, poor PrEP adherence during the pandemic was found among participants with likely less access to MSM-specific HIV care, such as MSM married or cohabitating with a woman and MSM who had not been tested for HIV in the last month. To improve PrEP adherence, public health officials must improve outreach to these subpopulations, such as sending at-home HIV tests, re-inforcing risks for HIV acquisition if poor adherence to PrEP, and if poor adherence is likely to continue, suggesting stopping PrEP use to prevent drug resistance. Knowledge about challenges to PrEP use due to COVID-19 provides first-hand real-world evidence of issues that must be addressed prior to wide-spread PrEP implementation globally.

In this study, similar to peer studies finding decreases in the number of sexual partners and sexual practices since the onset of COVID-19 (10, 14, 17), we observed that 93.5% of Chinese MSM PrEP users were impacted by lockdown restrictions and had fewer sexual partners and fewer sexual acts. These decreases may suggest that our participants overall may have temporarily lower risks of HIV infection. Nevertheless, no excess risks among MSM PrEP users were found in developing countries before. However, compared with those sexually active individuals before the pandemic, among those who were sexually active during the pandemic, a much higher percentage had CAI with sexual partners. PrEP cannot be used to replace condoms completely and is a part of a comprehensive prevention strategy that includes counseling and behavioral interventions promoting condom use, abstinence, and monogamy (18, 19). An online survey among MSM from the United States found conflicting results and did not find an effect on condom use during COVID-19 (20). A possible explanation is that China was the first country impacted by COVID-19 and quickly introduced physical distancing restrictions and lockdowns, leading to sudden significant changes in lifestyle and sexual practices, while other countries could have anticipated potential restrictions based on news from China. In the future, public health officials must target individuals who are likely to maintain sexual activity during times where general sexual activity drops and promote safer sex, such as high PrEP adherence and condom use. In addition, we strongly recommend that medical staff and community volunteers popularize safety knowledge regularly and emphasize the significance of PrEP good adherence.

As suboptimal PrEP adherence will decrease the effectiveness of PrEP (21), hence, all global public health sections highlight the importance of maintaining high medication adherence among subjects who take oral PrEP pills before or after the COVID-19 era. We found worse adherence during COVID-19 as more than one-third (35.7%) of participants did not obtain PrEP refills, and a half (50.1%) self-reported missing at least one PrEP dose in the previous month. Our findings are similar to the increase in the proportion who discontinued PrEP use and the increase in the mean number of missed PrEP doses found among MSM in the Southern United States (22). Individuals may have discontinued PrEP use because they were no longer engaging in sexual activity since COVID-19. Among those still engaging in sexual activity, they could have been unable to access PrEP and thus had worse adherence because they could not access PrEP or PrEP support when facilities were closed due to lockdowns. If users discontinued using PrEP, public health officials should reach out frequently to see if they would like to begin using PrEP again, and if users had worse adherence to PrEP, practitioners should ensure access to medication through a no-contact method, such as the mail and counsel users on the importance of adherence to prevent HIV transmission.

Frequent HIV testing can also help prevent HIV transmission, but during the COVID-19 pandemic, the rate of HIV testing was substantially reduced and correlated with poor PrEP adherence among Chinese MSM PrEP users. Our finding of less HIV testing, both HIVST and facility-based HIV-testing, was similar to results from other studies (23, 24). An online survey from the Southern United States also found that one-quarter of MSM PrEP users encountered obstacles to HIV testing (22). These difficulties in accessing HIV testing could have been caused by the physical distancing restrictions in response to COVID-19 and thus restricting access to facility-based HIV testing. However, continuous use of PrEP pills when infected with HIV can lead to HIV drug resistance events (25, 26), and PrEP users must be tested for HIV at frequent intervals. More web-based health promotion (e.g., text messaging) and home-based HIV-testing (e.g., oral self-tests) were practical tools for raising awareness of sexual health and HIV-testing in the United Kingdom and Africa (27, 28). Thus, to flatten the curve of the COVID-19 pandemic, we suggest providing counseling and HIVST through a no-contact method to PrEP users to ensure timely knowledge of HIV serostatus and prevent HIV drug resistance.

Along with decreased HIV testing, using condoms consistently with regular male sexual partners was significantly correlated with increased odds of poor PrEP adherence during COVID-19. Consistent with our results, low PrEP adherence in East Africa was associated with using condoms with all types of partners (29). We additionally found a decrease in self-perceived risk of HIV acquisition among PrEP users during COVID-19, which may be from decreased sexual activity or using condoms consistently with regular male sexual partners.

In addition to consistent condom use with regular male sexual partners, being married or cohabitating with a woman was also correlated with poor PrEP adherence during COVID-19. Due to traditional Chinese culture and attitudes toward homosexuality, up to 25–35% of Chinese MSM have already been in a heterosexual marriage and more than 70% of MSM may eventually form a family with a woman to have children and help conceal their homosexuality (30). Similarly, MSM partnering with a woman for cultural reasons also occurs in Nigeria and India (31, 32). However, despite this common practice, little is known about MSM who are married or cohabiting with a woman and their adherence to PrEP. It is possible that these partnered MSM feared disclosure of their PrEP use and, thus, sexual orientation, leading to poor PrEP adherence. Discrete yet effective strategies are needed to improve PrEP adherence among MSM married or cohabitating with a woman.

### Strengths and Limitations

By surveying changes in PrEP adherence and HIV-related behaviors from before to during the COVID-19 pandemic from a large population of Chinese MSM who used PrEP in four Chinese cities, our results were more representative. We were additionally able to stratify differences in adherence during the COVID-19 by PrEP regimen and found no difference in adherence between them. By being the first study to identify changes in PrEP adherence and HIV-related behaviors among Chinese MSM from before to during the COVID-19 pandemic, we were able to determine obstacles to PrEP adherence due to lockdown that must be addressed in possible future societal restrictions. A limitation of this study was that it used self-reported measures to define PrEP adherence. Another limitation was that we asked sensitive questions about sexual activities and our data may have been influenced by social desirability bias. To address this issue, the survey was conducted online, was self-reported, and was anonymous.

## Conclusion

We demonstrated a substantial increase in poor adherence and CAI among those sexually active, and a decrease in HIV testing among Chinese MSM who used PrEP from before to during the COVID-19 pandemic. Our results suggest that some MSM PrEP users have a likely increased risk of HIV acquisition during COVID-19, and health workers should develop online targeted interventions, such as adding online follow-up, promoting safer sex, PrEP adherence, and condom use, providing no-contact counseling and HIVST to increase knowledge of HIV serostatus, and prevent drug resistance event to PrEP, and discrete strategies to reach out to MSM married or living with a woman to promote PrEP adherence.

## Data Availability Statement

The datasets presented in this study can be found in online repositories. The names of the repository/repositories and accession number(s) can be found in the article/Supplementary Material.

## Ethics Statement

This study was reviewed and approved by the Medical Science Research Ethics Committee of the First Affiliated Hospital of China Medical University ([2018]2015-139-5) and was registered with the Chinese Clinical Trial Registry (ChiCTR-IIN-17013762). The patients/participants provided their written informed consent to participate in this study.

## CROPrEP Study Team

Members of the CROPrEP study team not included in the authors' list include, Xiaoqing He, Yao Li, Fang Zhao, Yijun Duan, Rui Li, Shangcao Li, Hang Li, Zhili Hu, Rantong Bao, Sitong Cui, Zhaozhen Liu, Zehao Ye, and Xiaoyun Shi.

## Author Contributions

YG conceived and designed the study and analyzed the data. LJ, QH, HoW, XH, YC, HuW, LZ and ZC performed the study. YG, QH, and SL draw the figures and tables. YG, QH, ZW, and SL wrote and revised the manuscript. All authors reviewed and approved the final manuscript.

## Funding

This study was supported by the Mega-Projects of National Science Research for the 13th Five-Year Plan [2017ZX10201101], the National Natural Science Foundation of China [81872674], and the National Science and Technology Major Project [2018ZX10101001-001-003].

## Conflict of Interest

Gilead sciences inc. donated all the PrEP drugs for the CROPrEP. The authors declare that the research was conducted in the absence of any commercial or financial relationships that could be construed as a potential conflict of interest.

## Publisher's Note

All claims expressed in this article are solely those of the authors and do not necessarily represent those of their affiliated organizations, or those of the publisher, the editors and the reviewers. Any product that may be evaluated in this article, or claim that may be made by its manufacturer, is not guaranteed or endorsed by the publisher.

## Abbreviations

COVID-19: coronavirus disease 2019
PrEP: pre-exposure prophylaxis
MSM: men who have sex with men
OR: odds ratio
CI: confidence interval
CAI: condomless anal intercourse
WHO: World Health Organization
PY: person-years
TDF/FTC: tenofovir/emtricitabine
CROPrEP: the China Real-world Oral Intake of PrEP
IQR: interquartile range
HIVST: HIV self-testing
aOR: adjusted odds ratio.
