# Supplementary material for: Medication Non-adherence and Condomless Anal Intercourse Increased Substantially During the COVID-19 Pandemic Among MSM PrEP Users: A Retrospective Cohort Study in Four Chinese Metropolises
Source: Front Med (Lausanne). 2022 Apr 29;9:738541. doi: 10.3389/fmed.2022.738541 (PMC9100828; doi:10.3389/fmed.2022.738541)
Supplement: Supplementary file 1 [file Data_Sheet_1.docx]

**
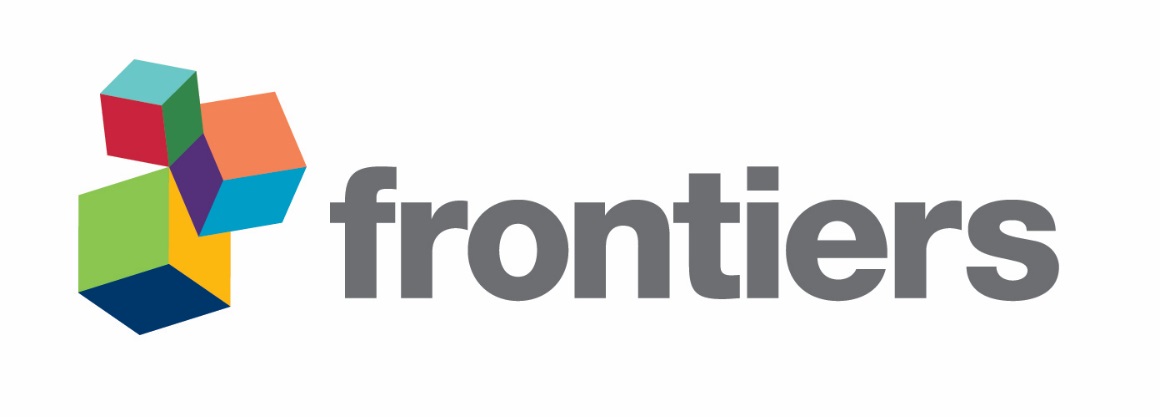
**

**Table of Contents**

**Supplementary Appendix 1:** **Study protocol**

Supplementary Appendix 2: Questionnaire

Supplementary Appendix 3: STROBE Statement

This appendix formed part of the original submission and has been peer-reviewed

**Study protocol**

**Protocol for medication non-adherence and condomless anal intercourse increased substantially during the COVID-19 pandemic among MSM PrEP users: A retrospective cohort study in four Chinese metropolises**

**Background**

Since the declaration of the coronavirus-19 (COVID-19) pandemic by the World Health Organization (WHO) on March 11, 2020, social distancing has interrupted hospital-based HIV prevention methods, HIV care, and testing services, particularly among men who have sex with men (MSM) (1,2).

Almost one quarter (23%) of new HIV infections globally in 2019 were among MSM, and the percentage of incident HIV infections among MSM was even higher in the Asia-Pacific region (3). The HIV incidence among Chinese MSM has increased from 3·24 new infections per 100 person-years (PY) in 2005–2008 to 5·50 new infections per 100 PY in 2012–2014 (4). New strategies to prevent HIV transmission, especially those that are effective despite social distancing, are needed to address the increasing HIV epidemic among Chinese MSM.

Pre-exposure prophylaxis (PrEP) is an innovative and effective biomedical HIV prevention strategy for people at high risk of HIV infection (5). PrEP is medication that is taken daily or event-driven to prevent HIV transmission and is frequently tenofovir/emtricitabine (TDF/FTC). If used with optimal adherence, PrEP is highly effective at preventing HIV transmission (6,7). However, if adherence is less than 40%, PrEP is no longer protective towards HIV transmission (8). Since the onset of the COVID-19 pandemic, few studies have assessed PrEP adherence, but several have reported that the number of PrEP users has decreased significantly (9,10). In the United Kingdom, Belgium, and Australia, there have been 80·0%, 47·0%, and 41·8% reductions in HIV PrEP users after the outbreak of COVID-19, respectively (11-13). In addition to decreasing use of PrEP, changes in HIV-related sexual behaviors and increased barriers to accessing HIV prevention and testing services during the COVID-19 pandemic could lead to an increase in HIV acquisition (9,11,14). Little is known about possible changes in PrEP adherence and HIV-related sexual and testing behaviors from before to during the COVID-19 pandemic.

We thus investigated the impact of the COVID-19 pandemic on PrEP adherence and HIV-related sexual and testing behaviors among Chinese MSM PrEP users and determined factors correlated with poor adherence to PrEP during the COVID-19 outbreak. Our findings will help researchers develop interventions to maintain and support PrEP use during the pandemic.

**Research objectives**

We did the survey to access the impact of the COVID-19 crisis and related restrictions on PrEP adherence and HIV risk behaviour changes among Chinese MSM PrEP users. Additionally, we also investigated to find the corrected factors with poor PrEP adherence to provide improvement strategies and recommendations

**Study setting**

The study includes four study sites (First affiliated hospital of China Medical University in Shenyang, Shenzhen Third People's Hospital in Shenzhen, Beijing Youan Hospital of Capital Medical University in Beijing, and Chongqing Public Health Medical Treatment Centre in Chongqing) in mainland China. We conducted this study from four individual sites through each ‘WeChat’ platform and phone calls according to the same study protocol. Project training was provided to all the related investigators before the study.

**Study design**

This study will be based on the CROPrEP project, an ongoing multicentre, real-world trial of HIV PrEP to assess effectiveness, adherence, and safety by two different PrEP dosing regimens, which have 500 MSM participants separately in four major cities in China (Shenyang, Beijing, Shenzhen, and Chongqing) (15). We conducted a self-reported online survey to access the changes in PrEP adherence and HIV risk behaviours before and during the COVID-19 pandemic among MSM PrEP users, enrolled via the Chinese social networking platform ‘WeChat’ and phone calls one by one. Additionally, we analysed the associated factors of poor adherence (Figure 1).

**Participant eligibility and enrolment**

The study participants were enrolled in the CROPrEP study.

The inclusion criteria were as follows:

- MSM aged 18 to 60 years.
- HIV negative since the previous HIV testing.
- Under the CROPrEP follow-up and completed the 4^th^-week follow-up survey.
- The follow-up was less than 48 weeks.
- Agree to participate in the study and voluntarily sign the informed consent.

The exclusion criteria were as follows:

- Erroneous personal identification (PID) code information.
- Discontinued or lost to follow-up.
- Changed HIV serostatus from negative to positive.
- Completed 48 weeks of follow-up.
- Miss the 4^th^-week follow-up survey.

**sample size**

The sample size was estimated based on our pilot study using PASS v15.0.5 software (NCSS Statistical Software, Kaysville, UT, USA). The rates of poor adherence in the study population before the COVID-19 pandemic were around 23% and were predicted to increase by about 31% during the pandemic. For the primary outcome with 90% statistical power and a type I error probability of 0.05, we conservatively estimated a sample size of 646 for this study. Assuming a rejection rate of 15%, a sample size of at least 760 respondents was required.


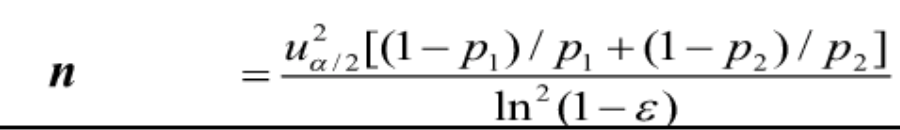


**Definitions and outcomes**

We defined regular partners as those in a stable relationship that did not engage in transactional sex; casual male partners were not in a stable relationship that did not involve transactional sex. Sexualized drug use was defined as using any of the following drugs in the previous month: rush poppers (alkyl nitrites), marijuana, and powdered cocaine. HIV testing experience included HIV testing in hospitals, VCT, or HIVST.

PrEP adherence was the primary study outcome. The self–reported PrEP adherence outcomes were expressed as a two-category variable (good or poor), based on self-reported missed PrEP doses, different PrEP intake regiments, and sexual behaviours during the COVID-19 pandemic (16). First, missing a dose of PrEP in the once-daily group or missing a dose of PrEP in the event-driven group (if they engaged in sexual behaviour during the previous one month) was defined as poor adherence^16^. Participants who took PrEP consistently in the once-daily group or did not take PrEP in the event-driven group due to the absence of sexual behaviours were defined as good adherence.

**Data collection and quality assurance**

**Questionnaire**

We had two rounds of similar questionnaires during the 4^th^-week follow-up and COVID-19. These sociodemographic and behavioural information for the previous month included the impact of COVID-19 (lockdown restrictions in response to COVID-19); HIV-risk behaviours (location for seeking homosexual partners; the numbers and types of sexual partners; frequency of sexual behaviours; condomless anal intercourse in the previous month; and sexualized drug use in the previous month); PrEP adherence (PrEP use and self-reported missed PrEP dose in the previous month); and HIV testing behaviours (recent HIV test in the previous month). Adaptive questions were used to reduce the number and complexity of the questions, and answers were submitted only after filling out items. Participants were permitted access to the same internet protocol address only once within a month. Before implementation, a pilot study was conducted using 45 MSM volunteers, epidemiologists, and clinicians to improve the study procedures and questionnaire.

**Ethical considerations**

This study was reviewed and approved by the Medical Science Research Ethics Committee of the First Affiliated Hospital of China Medical University and was registered with ChiCTR-IIN-17013762 (Chinese Clinical Trial Registry). Eligible participants completed the questionnaire after signing the informed consent voluntarily. Every participant had one chance to review and change their answers. All anonymous data and informed consent were supervised by an independent expert with access rights to improve information management. Each participant received $4.20 (30 yuan) as transportation compensation after they finished, the investigators qualified the questionnaire.

**Statistical analysis**

We examined the demographic and HIV-related behavioural characteristics of MSM PrEP users using descriptive statistical analysis such as frequencies and percentages. We compared the diﬀerence of adherence to PrEP, sexual behaviour, and HIV-testing before and during the COVID-19 pandemic by both the odds ratio and 95% CIs (GEE model). Univariable and multivariable logistic regression models were performed to assessed associated factors with poor adherence among MSM users during the COVID-19 pandemic. A two-tailed p-value below 0·050 was considered significant, and the p-value below 0·010 was considered marginal significance.^23^

**Study results and practical implications**

This study conducted in China, a developing country, showed a substantial increase in poor PrEP adherence, CAI, and lack of HIV testing during the COVID-19 crisis, reflecting some MSM PrEP users are more likely to engage in sexual acts even in times of COVID-19 threat. This evidence may help governments and health workers well understood gaps in the PrEP adherence and provide targeted comprehensive strategies to improve PrEP adherence, condom use, and regular HIV testing, such as online interventions, promotion of condom use, and HIVST reagents to MSM taking PrEP, especially for the subpopulation married or cohabitated with women.

**Abbreviations**

SARS-CoV-2: 2019 novel coronavirus

COVID-19: Coronavirus disease 2019

MSM: Men who have sex with men

PrEP: Pre-exposure prophylaxis

TDF/FTC: emtricitabine/tenofovir disoproxil fumarate

IP: Internet Protocol

HIVST: HIV self-testing

# References

1. World Health Organization. WHO announces the COVID-19 outbreak a pandemic (2020). http://www.euro.who.int/en/health-topics/health-emergencies/coronavirus-covid-19/news/news/2020/3/who-announces-covid-19-outbreak-a-pandemic,2020. [Accessed Nov 26, 2020]

2. Jiang H, Zhou Y, Tang W. Maintaining HIV care during the COVID-19 pandemic. The Lancet HIV. (2020). doi: 10.1016/S2352-3018(20)30105-3

3. Joint United Nations Programme on HIV/AIDS, 2020 DATA **(**2020). https://aidsinfo.unaids.org/. [Accessed Nov 26, 2020]

4. Joint United Nations Programme on HIV/AIDS, UNAIDS epidemiological estimates (2020). <https://aidsinfo.unaids.org/>. [Accessed Sep 29, 2020]

5. McCormack S, Dunn DT, Desai M, Dolling D. I, Gafos M, Gilson R.et al. Pre-exposure prophylaxis to prevent the acquisition of HIV-1 infection (PROUD): effectiveness results from the pilot phase of a pragmatic open-label randomised trial. The Lancet .(2016) 387: 53–60. doi: 10.1016/s0140-6736(15)00056-2

6. Molina JM, Capitant C, Spire B, Pialoux G, Cotte L, Charreau I,et al. On-Demand Preexposure Prophylaxis in Men at High Risk for HIV-1 Infection. N Engl J Med .(2015) 373: 2237–2246. doi: 10.1056/NEJMoa1506273

7. Molina JM, Charreau I, Spire B, Cotte L, Chas J, Capitant C, et al. Efficacy, safety, and effect on sexual behaviour of on-demand pre-exposure prophylaxis for HIV in men who have sex with men: an observational cohort study. The Lancet HIV. (2020). doi: 10.1016/S2352-3018(17)30089-9

8. Marrazzo JM, Ramjee G, Richardson BA, Gomez K, Mgodi N, Nair G, et al. Tenofovir-based preexposure prophylaxis for HIV infection among African women. N Engl J Med . (2015) 372: 509–518. doi: 10.1056/NEJMoa1402269

9. Hammoud MA, Grulich A, Holt M, Maher L, Murphy D, Jin, F, et al. Substantial Decline in Use of HIV Preexposure Prophylaxis Following Introduction of COVID-19 Physical Distancing Restrictions in Australia: Results from a Prospective Observational Study of Gay and Bisexual Men. J Acquir Immune Defic Syndr. (2021), 86: 22–30. doi: 10.1097/qai.0000000000002514

10. Davey DLJ, Bekker LG, Mashele N, Gorbach P, Coates TJ, Myer L. PrEP retention and prescriptions for pregnant women during COVID-19 lockdown in South Africa. The Lancet HIV. (2020). doi: 10.1016/S2352-3018(20)30226-5.

11. Junejo M, Girometti N, McOwan A, Whitlock G. HIV postexposure prophylaxis during COVID-19. The Lancet HIV. (2020). doi: 10.1016/S2352-3018(20)30146-6. 7: e460.

12. Reyniers T, Rotsaert A, Thunissen E, Buffel V, Masquillier C, Van Landeghem E, et al. Reduced sexual contacts with non-steady partners and less PrEP use among MSM in Belgium during the first weeks of the COVID-19 lockdown: results of an online survey. Sex Transm Infect. (2020). doi: 10.1136/sextrans-2020-054756

13. Charre C, Icard V, Pradat P, Brochier C, Lina B, Chidiac C, et al. Coronavirus disease 2019 attack rate in HIV-infected patients and in preexposure prophylaxis users. AIDS. (2020) 34: 1765–1770.doi: 10.1097/qad.0000000000002639

14. Chow EPF, Hocking JS, Ong JJ, Schmidt T, Buchanan A, Rodriguez E, et al. Changing the Use of HIV Pre-exposure Prophylaxis Among Men Who Have Sex With Men During the COVID-19 Pandemic in Melbourne, Australia. Open Forum Infect Dis. (2020).doi: 10.1093/ofid/ofaa275

15. Wang H, Zhang Y, Mei Z, Jia Y, Leuba SI, Zhang J, et al. Protocol for a multicenter, real-world study of HIV pre-exposure prophylaxis among men who have sex with men in China (CROPrEP). BMC Infect Dis. (2019) 19: 721. doi: 10.1186/s12879-019-4355-y

16. Riddell J, Amico KR, Mayer KH. HIV Preexposure Prophylaxis: A Review. JAMA. (2018) 319: 1261–1268. doi: 10.1001/jama.2018.1917
