# Supplementary material for: Medication Non-adherence and Condomless Anal Intercourse Increased Substantially During the COVID-19 Pandemic Among MSM PrEP Users: A Retrospective Cohort Study in Four Chinese Metropolises
Source: Front Med (Lausanne). 2022 Apr 29;9:738541. doi: 10.3389/fmed.2022.738541 (PMC9100828; doi:10.3389/fmed.2022.738541)
Supplement: Supplementary file 2 [file Data_Sheet_2.doc]

**
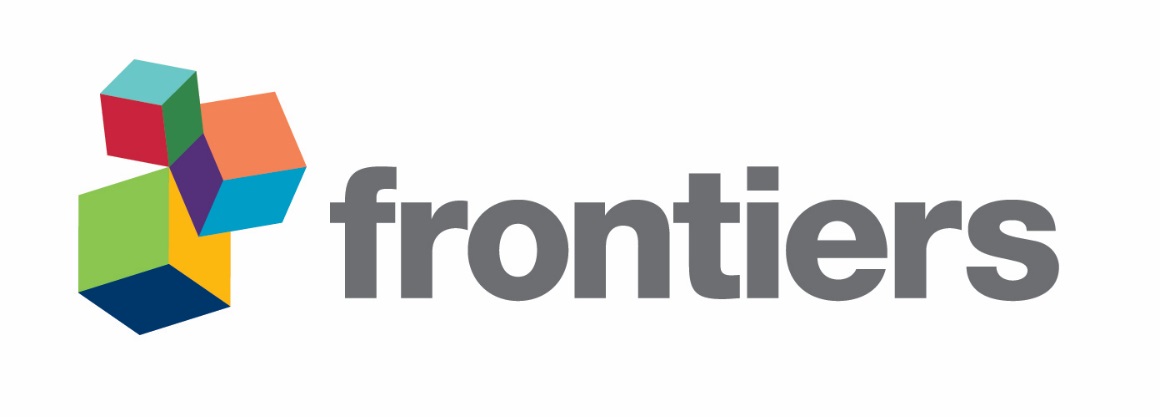
**

**Supplementary** **Appendix 2: Questionnaire**

**Behavior and adherence to PrEP survey of PrEP participants during COVID-19 pandemic**

Hello, dear friends! In order to timely understand the impact of the sudden outbreak of the coronavirus disease 2019 (COVID-19) on the behavior, HIV testing services and status of PrEP taking among men who have sex with men (MSM) population, providing higher quality HIV and COVID - 19 with the guidance of medical information, we sincerely hope you could spare 5-10 minutes to complete the following questionnaire. After completing the survey, we will provide a subsidy of about $4.20 per person. This survey is anonymous, and your answer will be kept strictly confidential, thank you for your support!

**Part 1: Socio-demographic characteristics**

1-1 Your PID number:

1-2 Your age:

1-3 Highest education attained:

1. Primary school or lower
2. Junior high school
3. High school
4. College
5. Postgraduate degree and above

1-4 Monthly personal income (RMB):

1-5 Initial PrEP dosing regimens:

1. Once-daily regimen
2. Event-driven regimen

1-6 Marital status now:

(1) Never married

(2) Married or cohabitating with female

(3) Cohabitating with male

(4) Divorced or separated

(5) Widowed

1-7 What's your sexual orientation?

1. Homosexual
2. Bisexual
3. Heterosexuality
4. Not sure

1-8 What are the following lockdown restrictions to restrict the movement of people in your area in response to COVID-19?

1. The city has already been closed
2. Suspension of transportation lines such as buses and subways
3. The travel is prohibited or restricted
4. Other restrictions
5. No restrictions

1-9 What is your current work/life status?

1. Have returned to work
2. Delayed returned to work/school
3. Being isolated at home
4. Being medical isolated as close contacts
5. Being treated for COVID-19
6. Unemployed

1-10 Are you concerned about the COVID-19 outbreak?

1. Always
2. Often
3. Sometimes
4. Little
5. Never

**Part 2: HIV-related risk behaviors**

- 1. What’s your primary venue to seek homosexual partners in the past month?

1. Geosocial networking for gays (Blued, Jack’d, etc.)
2. Popular social software (WeChat, QQ, etc.)
3. Park, bathroom, or public toilets
4. Club
5. Having regular sexual partners
6. No sexual partner
   1. In the past month, how often have you had sexual acts?
7. Almost every day
8. Every week
9. Less than once a week
10. No sex (Skip to 2-14)
    1. How many times do you have sex per week on average?
    2. In the past month, what was your primary sexual role with male?
11. “1” (insertive anal intercourse)
12. “0” (receptive anal intercourse)
13. “0.5” (both insertive and receptive anal intercourse)
14. Oral intercourse only (69)
    1. What type of male partner did you have in the last month? (multiple choice)
15. Regular male sex partners
16. Casual male sex partners
17. Commercial sex partners

****Note:*** Regular partners are those who were in a stable relationship without transactional sex in the past one months; Casual partners are those who were not in a stable relationship, had no cash or kind payment. Commercial sex refers to sexual behavior with money or material transactions, such as finding a money boy (MB) or doing MB yourself.

***Regular male sex partners*** *(only for those who were having regular male sex partners)*

- 1. In the past month, how many regular sexual partners have you had?
  2. In the past month, how often did you use condoms with regular sexual partners?

1. Every time
2. Most
3. Sometimes
4. Never
   1. Did you know the HIV status of regular sexual partners?
5. Yes
6. No

****Note:*** If you haven’t seen a recent HIV test result report for your partner with your own eyes, please select “No”.

***Causal male sex partners (only for those who were having causal male sex partners)***

- 1. How many casual sexual partners have you had?
  2. In the past month, how often did you use condoms with causal sexual partners?

1. Every time
2. Most
3. Sometimes
4. Never

***Commercial male sex partners*** *(only for those who were having commercial male sex partners)*

- 1. How many commercial sexual partners have you had?
  2. In the past month, how often did you use condoms with commercial sexual partners?

1. Every time
2. Most
3. Sometimes
4. Never
   1. In the past years, did you sex with HIV-positive male partners?
5. Yes
6. No
   1. In the past month, have you used the following chemsex-related drugs?
7. Inhalants (RUSH, popper, nitrogen, glue, olfactory oil, etc.)
8. Amphetamine-type stimulants (Dalibor, methamphetamine, etc.)
9. Ketamine
10. Cannabis (cannabis, cannabis buds, hemp grass, cannabis resin, etc.)
11. Tramadol/ Dextromethorphan Hydrobromide Tablets
12. Codeine phosphate
13. Hallucinogens (Ecstasy, Zero capsule, psychedelic mushrooms, trips, K powder, etc.)
14. 5-MeO-Dip
15. Nitrous oxide
16. Others
17. None of above
    1. In your opinion, your risk level of HIV infection is:
18. Very high risk (> 75%)
19. High risk (51% - 75%)
20. Moderate risk (25% - 50%)
21. Lower risk (< 25%)
22. No risk (0%)
    1. In the past month, did you have HIV testing?
23. Yes
24. No (Skip to 2-18)
    1. In the past month, which of the following methods have you used for HIV testing?
25. Voluntary Counseling and Testing (VCT)
26. Hospital
27. Center for Disease Control and Prevention (CDC)
28. Community organizations
29. HIV self-testing (HIVST)
30. Others
    1. In the past month, what was the change of the frequency of your HIV testing during COVID-19 pandemic？
31. Increase
32. Decrease
33. No change

**Part 3: Adherence to PrEP**

- 1. In the past month, have you taken PrEP (Truvada, Emtricitabine and Tenofovir Disoproxil Fumarate Tablets)?

1. Yes
2. No
   1. In the past month, have you missed PrEP dose?

(1) Yes

(2) No (Skip to 3-4)

- 1. In the past month, how many times did you missed PrEP dose?
  2. In the past months, on average, how would you rate your ability to take all your PrEP drugs as your doctor prescribed?

1. Very poor
2. Poor
3. Fair
4. Good
5. Very good
6. Excellent

**(This is the end of the survey, thanks for your support!)**
